# Genome-wide comparative analysis revealed significant transcriptome changes in mice after *Toxoplasma gondii* infection

**DOI:** 10.1186/1756-3305-6-161

**Published:** 2013-06-04

**Authors:** Boyin Jia, Huijun Lu, Quan Liu, Jigang Yin, Ning Jiang, Qijun Chen

**Affiliations:** 1 Key Laboratory of Zoonosis, Institute of Zoonosis/College of Veterinary Medicine, Jilin University, Xi An Da Lu 5333, Changchun 130062, China; 2 MOH Key Laboratory of Systems Biology of Pathogens, Institute of Pathogen Biology, Chinese Academy of Medical Sciences & Peking Union Medical College, Beijing, Dongdan Santiao 9, Beijing 100730, China; 3 Institute of Military Veterinary, Academy of Military Medical Sciences, Key Laboratory of Jilin Province for Zoonosis Prevention and Control, 666 Liuying Xilu, Changchun 130122, Jilin Province, China

**Keywords:** *Toxoplasma gondii*, RH strain, ME49 strain, Microarray, Gene ontology and pathway analysis, Brain tissues, Peripheral lymphocytes

## Abstract

**Background:**

*Toxoplasma gondii* is an intracellular parasite that can modulate host responses and presumably host behavior. Host responses as well as pathogenesis vary depending on the parasite strains that are responsible for infection. In immune competent individuals, *T. gondii* preferentially infects tissues of the central nervous systems (CNS), which might be an additional factor in certain psychiatric disorders. While in immune-compromised individuals and pregnant women, the parasite can cause life-threatening infections. With the availability of the genome-wide investigation platform, the global responses in gene expression of the host after *T. gondii* infection can be systematically investigated.

**Methods:**

Total RNA of brain tissues and peripheral lymphocytes of BALB/C mice infected with RH and ME 49 strain *T. gondii* as well as that of healthy mice were purified and converted to cRNA with incorporated Cy5-CTP (experimental samples), or Cy3-CTP (control samples). The labeled cRNA probes were hybridized to the Whole Mouse Genome Microarray. The impact of parasite infection on gene expression in both brain tissues and peripheral lymphocytes were analyzed. Differentially expressed genes were revalidated with real-time quantitative reverse transcriptase-polymerase chain reaction (Q-PCR).

**Results:**

Data indicated that the genes associated with immunity were up-regulated after infection by the two parasite strains, but significant up-regulation was observed in both brain tissues and peripheral lymphocytes of mice infected with ME49 strain compared to that infected by RH strain. The pathways related to pathogenesis of the nervous system were more significantly up-regulated in mice infected with RH strain.

**Conclusions:**

Genetically distinct *T. gondii* strains showed clear differences in modulation of host pathophysiological and immunological responses in both brain tissue and peripheral lymphocytes. It was likely that some of the host responses to *T. gondii* infection were universal, but the immune response and CNS reaction were in a strain-specific manner.

## Background

*Toxoplasma gondii* is an obligatory intracellular parasite that causes diverse pathological effects in humans and other warm-blooded vertebrates [1,2]. *T. gondii* has an unusual clonal population structure which exists with limited genetic diversity but belongs to three distinct clonal lineages and displays markedly different levels of virulence in mice [3]. Type I strain, such as RH strain is considered as the most virulent in mice, and it has been frequently found in individuals at risk of atypical ocular toxoplasmosis [4]. Type II strain, like ME49 strain is less pathogenic with lower LD50 values than that of RH strain and this strain has been found in the majority of human infections [5]. Type III *T. gondii* is rarely found in humans, but the reason is unclear [6]. Approximately 80% of individuals are asymptomatic after *T. gondii* infection, partially due to effective innate responses [7]. *T. gondii* establishes parasitization in the host by crossing biological barriers such as gut epithelia, the placenta or the blood–brain barrier [8]. Studies have found that parasites of type I strains grow faster *in vitro* than that of type II or III strains [9].

Parasite specific CD4+ and CD8+ T cell-mediated immunity, a strong Th1-type response which is predominant in the immunocompetent host’s immune reaction, has been regarded as the main component in anti-Toxoplasma infection [10], meanwhile humoral immunity plays a supplementary role [11]. The combination of IL-12 and IFN-γwas proposed as the core of defence against the parasite in early infection [12]. Neutrophils, macrophages, NK cells and NKT cells are another elements involved in the immune response to *T. gondii* infection [13]. However, once the host’s immune system balance is broken, it would lead to serious consequences, such as in AIDS patients [14]. Missing or defectiveness in function of immune system will result in a large number of proliferation of tachyzoites, which will lead to a severe consequence to the host [12].

Microarray represents the first generation of analytical tools with the capacity of global gene expression profiling in both pathogen development and host-pathogen interactions [15]. It has been extensively used to identify alterations in gene expression of bacterial [16] and viral infection [17]. Microarray has also been used to investigate gene expression of parasites such as *Eimeria maxima* and *Plasmodium falciparum*[18,19]. In studies on *T. gondii* infections, microarray has been mainly applied to analyze cell-specific responses with cultivated cells such as human foreskin fibroblasts, macrophages, dendritic cells, Muller cells, rat retinal vascular endothelial cells [20-23]. Using a cDNA microarray, Fouts et al. compared the expression profiles of human foreskin fibroblast cells infected by bradyzoites and tachyzoites. It was found that more genes were up-regulated in the fast growing tachyzoite-infected cells [24]. Further studies with cultivated Muller cells have found that *T. gondii* could induce cell immune responses, which could not control the differentiation of the parasites [22]. In addition, Xiao et al. examined the transcriptional profile of human neuroepithelioma cells in response to infection of type I, II, III of *T. gondii* by using microarray analysis [25]. However, all these studies were performed with cell lines infected by *T. gondii*, whereas little is known about changes in hosts *in vivo*. Furthermore, several studies have indicated that parasite infection in the host neural cells may cause behavioral alterations, but no conclusive evidence has been identified [26-28]. More studies are still necessary in order to increase our understanding of host–parasite interactions in *T. gondii* infection. In this study, we systematically analyzed the transcriptomes in both brain tissues and peripheral lymphocytes in BALB/C mice after infection by Type I (RH strain) and II (ME49 strain) *T. gondii* respectively. We found that hosts displayed distinct neurological and immunological responses to the infection of the two strains, which may explain the pathological background of infection caused by genetically different *T. gondii* strains.

## Methods

### Parasites and mouse tissues

RH and ME49 strains of *T. gondii* were routinely kept in the laboratory by cell cultivation. Thirty female BALB/C mice (20–25 g) aged six to eight weeks were randomly divided into 3 groups. For collecting brain tissues and peripheral lymphocytes from infected mice, two groups of BALB/C mice (10 per group) were peritoneally infected with RH and ME49 strain with 10 tachyzoites per mouse respectively. Infection was confirmed by Giemsa staining of peritoneal fluid and PCR [29,30]. Brain tissues were collected from infected mice 8 days after infection. At first, the infected mice were exsanguinated and the blood was mixed with heparin for separation of peripheral lymphocytes. The mice were then soaked in 75% ethanol for 30 seconds. Whole brain tissues were harvested, rinsed extensively in PBS, and immediately frozen in liquid nitrogen.

Peripheral lymphocytes were purified using Lymphocyte Separation Medium (Lonza, NJ, USA). Briefly, the anticoagulated blood diluted 2× with Hank’s solution was carefully layed over the lymphocyte separation solution. Samples were centrifuged at 1500 rpm/min for 15 minutes at room temperature. The lymphocytes concentrated in the interphase (white layer) between the plasma and the separation solution were collected and washed with Hanks solution 3 times before further processing. The use of laboratory animals was reviewed and approved by the Ethical Committee of Jilin University, Changchun, China.

### RNA purification and microarray hybridization

Total RNA of brain tissues and peripheral lymphocytes from mice infected with RH and ME 49 strain *T. gondii,* as well as of healthy mice, was extracted using the Trizol reagent (Invitrogen, CA, USA) according to the manufacturer’s instruction. Purity and integrity of the RNA samples were assessed on the Agilent 2100 bioanalyzer with the RNA 6000 Nano LabChip reagent set (Agilent Technologies, China) as described [31]. The RNA was quantified spectrophotometrically and stored at −80°C before use.

Before microarray hybridization, two batches of total RNA were labeled, and hybridized to Agilent Whole Mouse Genome (4 × 44 K) Microarrays (one-color platform) (Agilent Technologies, Shanghai, China), which was comprised of 41,534 60-mer oligonucleotide probes representing over 41,000 mouse genes and transcripts. The array was designed to truly represent all known genes in the mouse genome and their resulting transcripts. Briefly, first strand cDNA was synthesized with a reverse transcription primer containing a T7 promoter sequence. cRNA was generated with T7 RNA polymerase, which simultaneously amplifies the target and incorporates Cy3- or Cy5-labeled CTP with at least a 100-fold RNA amplification rate. cRNA from of infected mice was labeled with Cy5-CTP, while cRNA from control samples was labeled with Cy3-CTP. The labeled cRNA samples were then fragmented in fragmentation buffer at 60°C for 30 min before hybridization. The slides were washed, stabilized and scanned with the Agilent Technologies Microarray Scanner (Agilent Technologies, Shanghai, China).

### Data acquisition and bioinformatic analysis

Signals from all hybridization reactions were acquired and quality-checked by filtration of unspecific signals. Eventually, only signals with an absolute value of a Log2 Red/Green Lowess Normalized Ratio were selected and further analyzed. Data from six subject pairs were acquired. We used Gene ontology analysis and Pathway analysis to identify which of these genes showed significantly differential expression.

Gene ontology analysis was applied to annotate the main function of the differentially expressed genes according to the key functional classification of genes in NCBI [32,33]. The main categories of Gene ontology were involved in the top biological functions: molecular and cellular functions, diseases and disorders, and physiological system development and function [34]. Generally, Fisher’s exact test and *χ*^2^ test were used to classify the Gene ontology category, and the false discovery rate (FDR) was calculated to correct the p-value. The smaller the FDR, the less possibility of the error in judging the p-value [35]. Similarly, pathway analysis was used to find out the significant pathways of the differentially expressed genes according to KEGG [36]. The ratio of genes from the data set that map to the pathway divided by the total number of genes that map to the standard pathway was displayed. Fischer’s exact test and *χ*^2^ test were used to calculate the corresponding p-value [37]. P-value < 0.05 and FDR < 0.05 were used as a threshold to select significant Gene ontology categories and Pathways [38].

### Data validation by quantitative real-time PCR

Genes with significant differences in expression in mice before and after infection by *T. gondii* identified in microarray were further validated with Q-PCR. The RNA templates were reverse transcribed to cDNA using 200 U AMV Reverse Transcriptase (Promega, CA, USA) according to the manufacturer’s instructions. Primers were designed to amplify sequences of 90–200 base pairs (bp) (Table 1). The 25 μl RT reaction contained 2 μg total RNA, 0.5 mM dNTP mix (Takara, Dalian, China), 200 U AMV Reverse Transcriptase, 40 U RNase inhibitor (Promega). Q-PCR reaction was performed using a 7500 Real-Time PCR System (Applied Biosystems, CA, USA) with SybrGreen PCR Master Mix (Applied Biosystems) according to the manufacturer’s instructions. The genes encoding GAPDH (glyceraldehyde-3-phosphate dehydrogenase) and β-actin were chosen as endogenous references. Relative expression was calculated using the comparative Ct method [39].

**Table 1:** Primers for selected genes analyzed using Q-PCR

**Genes**	**Primer sequence (5’-3’)**	**GenBank number**	**Length of PCR products (bp)**
Gnal	TGGGCAACAGCAGCAAGA	AC140336	131
	AAGCAGCAGGCGGTGAGT		
Itgb1	CTGGTCCCGACATCATCC	AC156608	81
	TCCAAATCAGCAGCAAGG		
C3	TGGACCAGACCGAACAGT	BC043338	125
	GAAGGCAGCATAGGCAGA		
Nos2	GAGCGAGTTGTGGATTGTC	AL592185	133
	CCAGGAAGTAGGTGAGGG		
Fgfr2	TCAGCTGGGGGCGCTTCATC	AC157606	158
	GGGGGCAACCACGTACGCTTC		
Fos	AGACCGTGTCAGGAGGCA	BC029814	111
	CCATCTTATTCCGTTCCCT		
Erbb4	ATGGCAGGTGCTATGGAC	AC167658	168
	GAAAGGTGGTTGGATTGT		
Stat5b	TGCTCATCAACAAGCCAGAC	AL591466	121
	AAAGGCATCAGATTCCAAA		
Igf1r	CCTTTGGGATGGTCTATGA	AC101879	88
	GCCTCGTTTACCGTCTTG		
Rac2	TGATGGTGGACAGTAAGCC	AL590144	122
	TAGCGAGAAGCAGATGAGAA		
Itgb2	TCGGCTTTGGGTCGTTTG	AC153830	151
	TGGTTGGAGTTGTCGGTTA		
Kdr	GCAAATACAACCCTTCA	BC020530	95
	ACCAATACCCTTTCCTCA		
Cblb	GCTCGGCTACAGAAATAC	AK147367	167
	TCCCTGCTACCATCAATC		
Cd4	ACTCACCCTCAAGATACCC	AC142254	119
	GAGCCACTTTCATCACCA		
Cd8a	ATTGGACTTCGCCTGTG	AC160090	109
	CTTTCGGCTCCTGTGGT		
Jun	ACGCCAACCTCAGCAACTTC	AK159196	193
	GTCTGCGGCTCTTCCTTC		
TNF	GTGGAACTGGCAGAAGAGG	CR974444	94
	CAAGCAGGAATGAGAAGAGG		
Mapk3	CGGATTGCTGACCCTGA	BC029712	111
	GGATTTGGTGTAGCCCTTG		
Ep300	CCAAGCATAGGGAATCAA	AC102262	184
	GGTCAGCAGAAGGAGCAG		
Prkca	AGTGCCAAGTTTGCTGTT	AL645535	143
	GGTAGGGCTTCCGTATGT		
GAPDH	AGGTCGGTGTGAACGGATTTG	BC082592	123
	TGTAGACCATGTAGTTGAGGTCA		
β-actin	CGTGAAAAGATGACCCAG	AC144818	167
	AGAGCATAGCCCTCGTAGA		

## Results

### Significant transcriptomic changes in mice infected by RH and ME49 strain *T. gondii*

Discriminant analysis demonstrated significant changes in the transcriptome of brain tissue and peripheral lymphocytes of mice before and after *T. gondii* infection of RH and ME49 strain (Table 2). Of the 41,174 genes presented in the mouse genome microarray, we identified 1,537 up-regulated and 1,213 down-regulated genes in brain tissues (Additional file 1), and 3,602 up-regulated and 4,045 down-regulated genes in peripheral lymphocytes in the group of mice as a result of infection by *T. gondii* RH strain, compared to healthy control samples (Additional file 2) (p ≤ 0.05). Similarly, we identified 1,842 up-regulated and 1,368 down-regulated genes in brain samples (Additional file 3) and 3,109 up-regulated and 2,777 down-regulated genes in peripheral lymphocytes in ME49 strain infected mice compared to healthy controls (Additional file 4). Furthermore, 1,179 up-regulated and 1,613 down-regulated genes in the brain tissue (Additional file 5), and 1, 605 up-regulated and 1, 417 down-regulated genes in peripheral lymphocytes (Additional file 6), were identified in mice infected with *T. gondii* RH strain compared to that infected with ME49 strain.

**Table 2:** **Number of genes showing up and down regulation in mice after infection by *T. gondii***

	**Up-regulation**		**Down-regulation**	
	**Brain**	**Periphery**	**Brain**	**Periphery**
RH strain VS Healthy	1,537	3,602	1,213	4,045
ME49 strain VS Healthy	1,842	3,109	1,368	2,777
RH strain VS ME49 strain	1,179	1,605	1,613	1,417

### Gene ontology and pathway analysis revealed distinct expression patterns in the brain tissues of mice infected by RH and ME49 strain *T. gondii*

**Figure 1:**
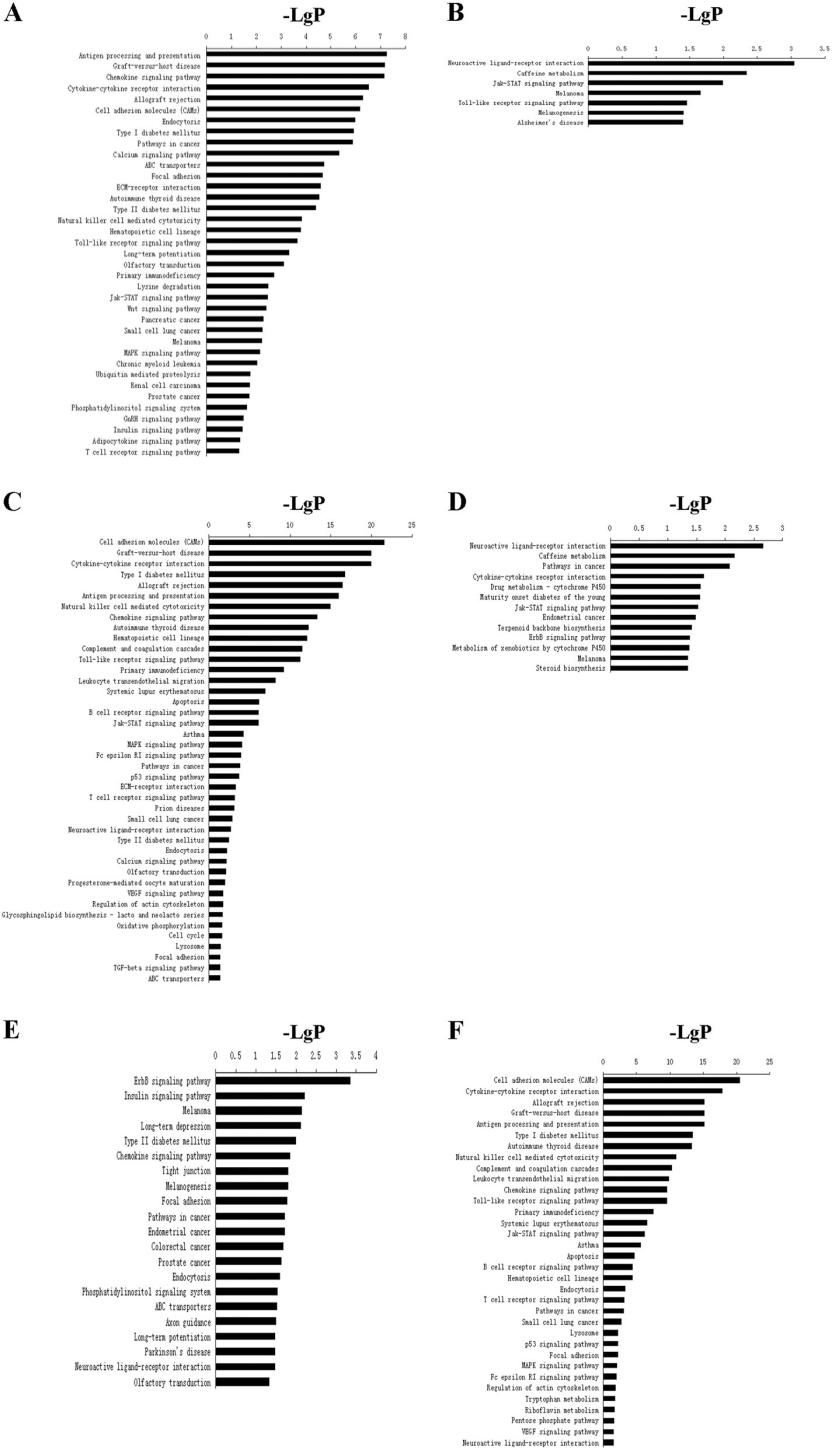
**KEGG pathway analysis for differentially expressed genes in the brain tissues of mice infected by RH and ME49 strain *T. gondii*.** (**A** and **B**) Pathways significantly up- and down-regulated in the brain tissues of mice infected by RH strain *T. gondii* compared to that of un-infected mice. (**C** and **D**) Pathways significantly up- and down-regulated in the brain tissues of mice infected by ME49 strain *T. gondii* compared to that of un-infected mice. (**E** and **F**) Pathways significantly up- and down-regulated in the brain tissues of mice infected by RH strain *T. gondii* compared to that of ME49 strain infected mice. P-value < 0.05 and FDR < 0.05 were used as a threshold to select significant KEGG pathways.

Genes differentially expressed in mice infected by RH and ME49 strain *T. gondii* were characterized with Gene ontology and pathway analysis to further categorize the functions. Comparing the transcriptome of mouse brains infected by RH strain *T. gondii* with that of healthy mice showed that genes involved in the response to stress were differentially expressed. However, the number of up-regulated genes was more than the number that were down-regulated, such as response to drug, response to hypoxia, response to ethanol and response to estradiol stimulus. Genes involved in immune responses including antigen processing and presentation of peptide antigen via MHC class I were over-presented among the up-regulated genes (Additional file 7: Figure S1A). On the other hand, genes involved in cell proliferation and adhesion including positive regulation of cell proliferation and regulation of epithelial cell proliferation were less presented among the down-regulated genes (Additional file 7: Figure S1B). In addition, in the brain tissue of RH infected mice, genes involved in neurological pathways such as focal adhesion, Wnt signaling and insulin signaling were up-regulated. The down-regulated genes were related to the nervous system including neuroactive ligand-receptor interaction and Alzheimer’s disease in the same group of mice (Figure 1A and B). In addition to the signaling pathway mentioned above, there were 12 up-regulated genes and 8 down-regulated genes in toll-like receptor signaling pathways in the brain tissue of mice infected by RH strain *T. gondii*. Similarly, there were 13 up-regulated genes and 12 down-regulated genes in Jak-STAT signaling pathway (Figure 1A and B).

The analysis of the transcriptome of the brains in mice infected by ME49 strain *T. gondii* as compared with that of healthy mice, showed that the most primary changes were found in expression of genes involved in inflammatory and immune responses, such as innate immune responses, antigen processing and presentation, positive regulation of I-kappaB kinase/NF-kappa B cascade including NF-kappa B transcription factor activity (Additional file 7: Figure S1C). On the other hand, the primary Gene Ontology categories for down regulated genes were related to the response to stress, such as responses to drugs and ethanol, hypoxia; responses to stimulus of glucose, estradiol and estrogen, glucocorticoid, and responses to lipopolysaccharide (Additional file 7: Figure S1D)*.* Thus, the responses to stress in the brain tissue of mice infected by ME49 strain were opposite to that in mice infected by RH strain.

Furthermore, in the brain tissue of mice infected by ME49 strain, differential expression in genes involved in immune pathways such as antigen processing and presentation, natural killer cell mediated cytotoxicity, T cell receptor signaling pathway, toll-like receptor signaling pathway and chemokine signaling pathway were more prominent. These pathways were differentially expressed and all of them were up-regulated in brain tissue of mice infected by ME49 strain *T. gondii*. In the genes involved in neurological pathways, 21 were up-regulated genes and 21 were down-regulated in neuroactive ligand-receptor interaction. In the genes involved in Jak-STAT signaling pathway, 21 genes were up-regulated and 12 genes were down-regulated (Figure 1C and D).

Further analysis carried out by comparing the expression in genes involving pathways in the brain tissue infected by RH strain *T. gondii* versus that by ME49 strain; showed that the number of down-regulated pathways was more than the number up-regulated. The main difference was in the pathways related to immune responses in the brain. The pathways of chemokine signaling pathway, toll-like receptor signaling pathway, Jak-STAT signaling pathway, B cell receptor signaling pathway and T cell receptor signaling pathway were all down-regulated in the brain of RH strain infected mice compared to that of ME49 strain infected mice. On the other hand, the genes in pathways related to neurological responses such as focal adhesion, insulin signaling and neuroactive ligand-receptor interaction were more up-regulated in the brain tissue of RH strain infected mice compared to that of ME49 strain infected mice (Figure 1E and F).

### Gene ontology and pathway analysis revealed distinct expression patterns in the peripheral lymphocytes of mice infected by RH and M49 strain *T. gondii*

**Figure 2:**
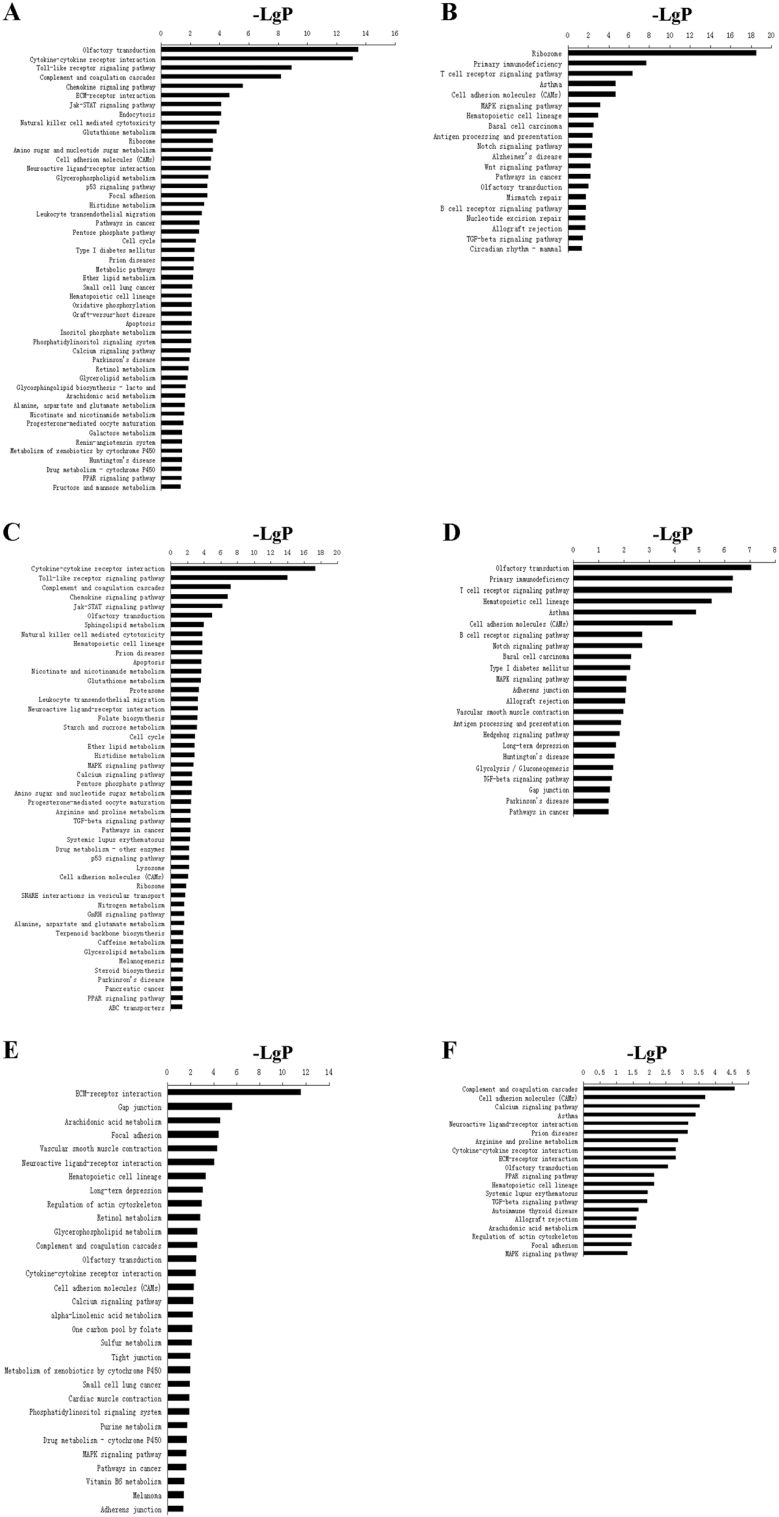
**KEGG pathway analysis for differentially expressed genes in KEGG pathway analysis for differentially expressed genes in peripheral lymphocytes of mice infected by RH and ME49 strain *T. gondii*.** (**A** and **B**) Pathways significantly up- and down-regulated in peripheral lymphocytes of mice infected by RH strain *T. gondii* compared to that of un-infected mice. (**C** and **D**) Pathways significantly up- and down-regulated in peripheral lymphocytes of mice infected by ME49 strain *T. gondii* compared to that of un-infected mice. (**E** and **F**) Pathways significantly up- and down-regulated in peripheral lymphocytes of mice infected by RH strain *T. gondii* compared to that of ME49 strain infected mice. P-value < 0.05 and FDR < 0.05 were used as a threshold to select significant KEGG pathways.

We further analyzed the transcriptomic changes in the peripheral lymphocytes in mice infected by RH and ME49 strain *T. gondii*. The transcriptional changes in lymphocytes in the RH strain-infected mice compared to that of uninfected mice were similar to that of the brain tissues (Additional file 8: Figure S2A and S2B). The most prominent up-regulated genes were involved in olfactory transduction, ribosome, cell adhesion molecules (CAMs), hematopoietic cell lineage related to metabolism. Genes involved in immune pathways such as toll-like receptor signaling pathways, chemokine signaling pathways, Jak-STAT signaling pathways and natural killer cell mediated cytotoxicity were moderately up-regulated. On the other hand, the pathways such as T cell receptor signaling pathways, antigen processing and presentation, and B cell receptor signaling pathways were all down-regulated compared to those in the healthy mice. Additionally, pathways of oxidative phosphorylation, sugar and glycerolipid metabolism involved in energy metabolism were up-regulated in the lymphocytes of RH strain-infected mice (Figure 2A and B).

Analysis in genes related to pathways in the peripheral lymphocytes in the ME49 strain *T. gondii* infected mice compared to those of healthy mice revealed that the number of significantly up-regulated pathways was more than those down-regulated, and the most up-regulated pathways were those involved in immune responses. In the peripheral lymphocytes in the ME49 strain *T. gondii* infected mice, the immune pathways such as cytokine-cytokine receptor interaction, toll-like receptor signaling pathway, chemokine signaling pathway, Jak-STAT signaling pathway and natural killer cell mediated cytotoxicity were prominently up-regulated (Figure 2C and D). On the other hand, pathways such as T cell receptor signaling pathway, B cell receptor signaling pathway, antigen processing and presentation were down-regulated. In addition, the neuroactive ligand-receptor interaction pathway related to the nervous system was up-regulated compared to that of healthy mice (Figure 2C and D).

However, the expression changes in the lymphocytes of mice infected by RH strain differed significantly from those infected by ME49 strain. The result of this analysis showed that the number of significantly up-regulated pathways was more than that of down-regulated pathways, and the main up-regulated pathways were those involved in metabolism, such as alpha-Linolenic acid metabolism, sulfur metabolism, metabolism of xenobiotics by cytochrome P450, vitamin B6 metabolism and so on. In addition to the signaling pathway, there were 14 up-regulated genes and 17 down-regulated genes in cell adhesion molecules (CAMs). Similarly, there were 24 up-regulated genes and 22 down-regulated genes in neuroactive ligand-receptor interaction (Figure 2E and F).

### Differentially expressed genes were confirmed by Q-PCR

**Figure 3:**
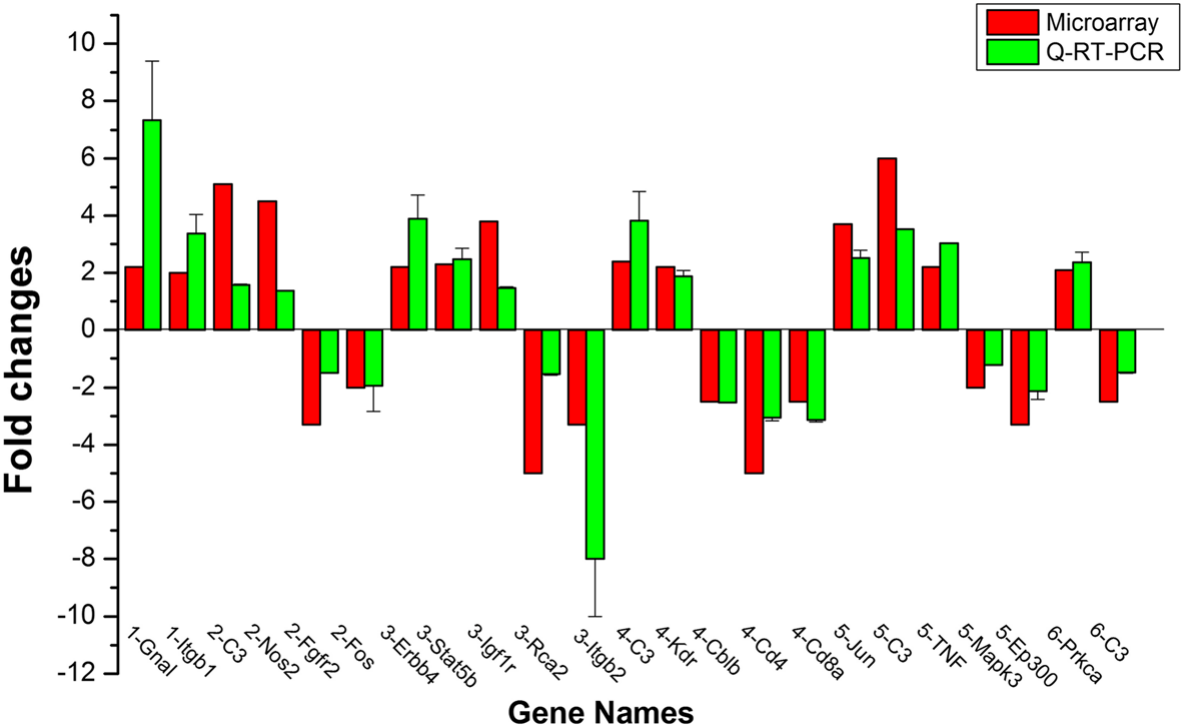
**Validation of up- and down-regulated expressed genes by Q-PCR.** The expression of 13 up-regulated genes such as Gnal, Itgb1, C3, Nos2, Erbb4, Stat5b, Igf1r, Kdr, Jun, TNF, Prkca and 10 down-regulated genes such as Fgfr2, Fos, Rca2, Itgb2, Cblb, Cd4, Cd8a, Mapk3, Ep300, C3 (C3 was regulated among ME49 strain brain versus control, RH strain peripheral lymphocytes versus control, ME49 strain peripheral lymphocytes versus control, RH strain peripheral lymphocytes versus ME49 strain peripheral lymphocytes) identified in microarray assay (in red color) was re-evaluated by Q-PCR (in green color). The Q-PCR results confirmed the data of the microarray.

Genes with significant differences in expression in mice before and after infection with *T. gondii*, that were identified in microarray were further validated with Q-PCR. Thirteen genes with up-regulation profiles and 10 genes with down-regulation profiles were selected (Table 1) and evaluated by Q-PCR. The results (Figure 3) confirmed the microarray data.

## Discussion

*Toxoplasma gondii* is an obligate intracellular parasite that can infect all nucleated cells. The parasite can not only cause severe pathogenesis to the host, but also actively manipulates host responses to benefit its proliferation and dissemination. The outcome following *T. gondii* infection depends on various factors, including host immune status and parasite factors. *T. gondii* mainly resides in the neuron cells during chronic infection, which might cause certain psychiatric disorders in the immune competent host [40,41]. To better understand the pathophysiology of toxoplasmosis, the global gene expression profiles in brain and peripheral lymphocytes following *T. gondii* infection were systematically investigated with a genome microarray platform in this study. Instead of simply reporting a list of differentially expressed genes, we sought to study the immune and neurological implications of gene expression changes in the context of biological pathways.

Previous studies have indicated that *T. gondii* induces responsive changes in protein expression in the brain tissue of infected mice [42]. Here, the global expression patterns in the brain tissue after infection by different phenotypic *T. gondii* strains were analyzed. The main differences in gene expression in the brain tissues in mice infected by RH strain and ME49 strain were in the aspects of neurological damage and immune responses. The genes involved in pathways related to neurological responses such as focal adhesion, insulin signaling and neuroactive ligand-receptor interaction were more up-regulated in the brain tissue of RH strain infected mice compared to that of ME49 strain infected mice (Figure 1). Contrary to the up-regulation of a large number of pathways, Alzheimer’s disease and neuroactive ligand-receptor interaction were the down-regulated pathways observed in the brain tissue of RH strain infected mice compared to that of un-infected mice. The primary features of Alzheimer’s disease (AD) have previously been described as a neurodegenerative disorder exhibiting intracellular neurofibrillary tangles and extracellular senile plaques [43]. Previous serological studies proved that *T. gondii* infection may be an aetiological factor associated with AD [44]. Jung et al. reported that *T. gondii* caused learning and memory disorder in Tg 2576 mice [45]. Down regulation of factors related to AD may reflect the ability of the parasite to inhibit cell apoptosis, which may maintain a better environment for the chronic parasitization in the host. Further, insulin signaling was another over-represented biological processes involved in neurological function. It was one of most important neurotrophic factors that seem to play a key role in the pathogenesis of AD [46]. For example, insulin signaling deficiency was related to AD pathology [47]. Thus, it is postulated that RH strain is prone to infect neuron cells and causes more brain damage than ME49 strain. Although the immune responses in the brains of mice infected by either RH or ME49 strain *T. gondii* were up-regulated (Figure 1), the genes involved in chemokine signaling pathway, toll-like receptor signaling pathway, Jak-STAT signaling pathway, B cell receptor signaling pathway and T cell receptor signaling pathway in the brain tissue of mice infected by ME49 strain compared to that of RH strain infected mice were significantly more up-regulated. Earlier studies have shown that TLR11 and MyD88 were required in the activation of early innate immune responses and mice which lack MyD88 were highly susceptible to *T. gondii* infection [48,49]. In this study, MyD88 was found to be significantly up-regulated in the mice infected by ME49 strain compared to that infected by RH strain. This may explain the pathological difference between the mice infected by the two toxoplasmal strains. Furthermore, the activation of the T cell receptor contributed to cellular immunity, which was conducive to protective immunity of humans and mice [50]. In this study, T cell receptor signaling pathway was found significantly up-regulated in the host infected with ME49 strain compared to that infected with RH strain. Thus it is likely that the innate and adaptive immune responses of the host were more strongly elicited by ME49 strain.

Peripheral lymphocytes are the most important immune components in responses to pathogen infection. However, the gene expression of immune cells during infections can be manipulated by the parasites. Here the transcriptomes of peripheral lymphocytes in mice infected by RH and ME49 strain were compared. The differences in gene expression in the peripheral lymphocytes in mice infected by RH strain and ME49 strain *T. gondii* were more significant than that in the brain tissues, especially with the genes involved in immune responses. In the peripheral lymphocytes in the ME49 strain *T. gondii* infected mice, the immune pathways such as cytokine-cytokine receptor interaction, toll-like receptor signaling pathway, chemokine signaling pathway, Jak-STAT signaling pathway and natural killer cell mediated cytotoxicity were prominently up-regulated compared to those of RH strain infected mice (Figure 2). Furthermore, we observed that the energy metabolism pathways were significantly up-regulated in mice infected by *T. gondii* RH strain compared to those infected by ME49 strain. This may be a reflection of the difference in proliferation rate between the two parasite strains. The fast-growing RH strain would cause more energy consumption in the host, and earlier studies have shown that oxidative phosphorylation was essential for maintaining the ATP level for the growing tachyzoite [51]. Our results also confirm the earlier findings. Furthermore, Weilhammer et al. reported that host cell glycolysis could promote the growth of tachyzoite and inhibit bradyzoite conversion [52]. Our data revealed that Glycolysis/Gluconeogenesis was down-regulated in mice infected by *T. gondii* ME49 strain, but there were no other significant changes in the energy metabolism in the host. This may indicate that the parasites were transforming from tachyzoite to the bradyzoite phase.

## Conclusions

The transcriptomic profiles of mice after infection by genetically distinct strains of *T. gondii* showed clear diversity in both brain tissues and peripheral lymphocytes. Gene ontology, pathway analysis indicated that the innate and adaptive response of the host was more strongly elicited in ME49 strain infected mice. Whereas, gene responses related to neurological disorders were prominent in RH strain *T. gondii* infected mice. With respect to the ability of *T. gondii* to influence host gene expression, it is likely that some of these effects were universal, while some of the genes involved in the immune response and CNS regulation of brain tissue and peripheral lymphocytes are strain-specific in manner. Future research should extend such studies to various types of immune response and brain cells between strains of different virulence effects on host gene expression.

## Competing interests

The authors declare that they have no competing interests.

## Authors’ contributions

BJ, QC and NJ conceived and participated in the design of the platform; BJ, HL, QL and JY carried out the experiments. The manuscript was drafted by BJ, QC and NJ. The final version was read and approved by all authors.

## Supplementary Material

Additional file 1 **Microarray hybridization results of up- and down-regulated genes in mouse brain after infection by RH strain *T. gondii*.** Click here for file

Additional file 2 **Microarray hybridization results of up- and down-regulated genes in mouse peripheral lymphocytes after infection by RH strain *T. gondii*.** Click here for file

Additional file 3 **Microarray hybridization results of up- and down-regulated genes in mouse brain after infection by ME49 strain *T. gondii*.** Click here for file

Additional file 4 **Microarray hybridization results of up- and down-regulated genes in mouse peripheral lymphocytes after infection by ME49 strain *T. gondii*.** Click here for file

Additional file 5 **Microarray hybridization results of up- and down-regulated genes in mouse brain tissues after infection by RH strain versus that infected by ME49 strain *T. gondii*.** Click here for file

Additional file 6 **Microarray hybridization results of up- and down-regulated genes in mouse peripheral lymphocytes after infection by RH strain versus that infected by ME49 strain *T. gondii*.** Click here for file

Additional file 7: Figure S1 GO category based on biological process for differentially expressed genes in brain tissues in mice infected by RH and ME49 strain *T. gondii*. (**A** and **B**) The significantly up- and down-regulated genes in the brain tissues of mice infected by RH strain *T. gondii* compared to that of un-infected mice. (**C** and **D**) The significantly up- and down-regulated genes in the brain tissues of mice infected by ME49 strain *T. gondii* compared to that of un-infected mice. *P*-value < 0.05 and FDR < 0.05 were used as a threshold to select significant GO categories. Click here for file

Additional file 8: Figure S2 GO category based on biological process for differentially expressed genes in peripheral lymphocytes in mice infected by RH and ME49 strain *T. gondii*. (**A** and **B**) The significantly up- and down-regulated genes in peripheral lymphocytes of mice infected by RH strain *T. gondii* compared to that of un-infected mice. (**C** and **D**) The significantly up- and down-regulated genes in peripheral lymphocytes of mice infected by ME49 strain *T. gondii* compared to that of un-infected mice. *P*-value < 0.05 and FDR < 0.05 were used as a threshold to select significant GO categories. Click here for file
